# Differences in early and late stages of information processing between slow versus fast participants

**DOI:** 10.1186/1755-7682-7-49

**Published:** 2014-11-24

**Authors:** Claudio Portella, Sergio Machado, Flávia Paes, Mauricio Cagy, Alexander T Sack, Ada Sandoval-Carrillo, Jose Salas-Pacheco, Adriana Cardoso Silva, Roberto Piedade, Pedro Ribeiro, Antonio Egídio Nardi, Oscar Arias-Carrión

**Affiliations:** Brain Mapping and Sensory Motor Integration, Institute of Psychiatry of Federal University of Rio de Janeiro (IPUB/UFRJ), Rio de Janeiro, Brazil; UNIABEU/Fisioterapia, Rio de Janeiro, Brazil; Panic & Respiration Laboratory, Institute of Psychiatry, Federal University of Rio de Janeiro, Rio de Janeiro, Brazil; Institute of Phylosophy (IFILO), Federal University of Uberlândia (UFU), Minas Gerais, Brazil; Physical Activity Neuroscience, Physical Activity Postgraduate Program, Salgado de Oliveira University (UNIVERSO), Niterói, Brazil; Quiropraxia Program of Faculty of Health Sciences, Central University (UCEN), Santiago, Chile; Biomedical Engineering, Federal University of Rio de Janeiro (COPPE/UFRJ), Rio de Janeiro, Brazil; Department of Cognitive Neuroscience, Faculty of Psychology and Neuroscience, Maastricht University, Maastricht, The Netherlands; Instituto de Investigación Científica, Universidad Juárez del Estado de Durango, Durango, México; School of Physical Education, Bioscience Department (EEFD/UFRJ), Rio de Janeiro, Brazil; Unidad de Trastornos del Movimiento y Sueño (TMS), Hospital General Dr. Manuel Gea González/IFC-UNAM, Mexico City, Mexico; Unidad de Trastornos del Movimiento y Sueño (TMS), Hospital General Ajusco Medio, Secretaría de Salud, Mexico City, Mexico; National Institute for Translational Medicine (INCT-TM), Rio de Janeiro, Brazil

**Keywords:** Slow- and fast-participants, Odd-ball, Event-related potential, P200, N200, Pre-motor potential, P300, Reaction time

## Abstract

The human brain is a system consisting of various interconnected neural networks, with functional specialization coexisting with functional integration occurring both; temporally and spatially at many levels. The current study ranked and compared fast and slow participants in processing information by assessing latency and amplitude of early and late Event-Related Potential (ERP) components, including P200, N200, Premotor Potential (PMP) and P300. In addition, the Reaction Time (RT) of participants was compared and related to the respective ERP components. For this purpose, twenty right-handed and healthy individuals were subjected to a classical ERP “Oddball” paradigm. Principal Component Analysis (PCA) and Discriminant Function analyses (DFA) used PRE components and the Reaction Time (RT) to classify individuals. Our results indicate that latencies of P200 (O2 electrode), N200 (O2), PMP (C3) and P300 (Pz) components are significantly reduced in the group of fast responding participants. In addition, the P200 amplitude is significantly increased in the group of fast responding participants. Based on these findings, we suggest that the ERP is able to detect even minimal impairments, in the processing of somatosensory information and cognitive and motor stages. Hence, the study of ERP might also be capable of assessing sensorimotor dysfunctions in healthy old-aged people and in neuropsychiatric patients (suffering from dementia, Parkinson’s disease, and other neurological disorders).

## Introduction

The human brain is a system consisting of various neural networks of multiple connectivity, with functional specialization coexisting with functional integration occurring both temporally and spatially at many levels [1]. Classical oddball paradigms, traditionally used in Event-Related Potential (ERP) studies, are ideal assessments of how the brain discriminates stimuli and processes probability, thereby being directly related to models of cognitive processing [2]. In a classical oddball paradigm, two stimuli are presented randomly, with one occurring infrequently. Participants are asked to discriminate a pre-defined target stimulus (frequent, 20%) from a non-target stimulus (infrequent, 80%).

ERP provides information such as the nature, organization, and timing of brain dynamics underlying sensory, perceptual, and cognitive processes. This information can be investigated and discussed through the calculation of the positive and negative voltage deflections in the brain’s electrical activity, which is evident in the averages of electroencephalography (EEG) epochs time-locked to a class of repeated stimuli or response events [2, 3]. ERP also provides the assessment of different stages of sensory information processing, indexing specific ERP components that reflect the primary and secondary processing of sensory input, the encoding, classification and guidance of the task, as well as the selection and response execution [4].

The early ERP components reflect basic sensory processing of stimuli at a lower level of processing, represented by P200 and N200 waves. In contrast, the later ERP components reflect the perceptual and cognitive processing of stimuli at a higher level of processing, represented by premotor potential (PMP) and the P300 wave [5]. Based on this rationale, in which the processing of sensory inputs plays a significant role for the cognitive and motor performance, the current study aimed to compare fast and slow responding participants by assessing both the latency and amplitude of early and late ERP components, including P200, N200, Premotor Potential (PMP) and P300 in the context of a visual oddball paradigm.

## Experimental procedures

### Participants

Twenty healthy participants (10 male and 10 female; mean age: 33.5, SD: 11.5) were recruited for the current study. All participants were right handed and had normal or corrected to normal vision [6]. Inclusion criteria were: absence of mental or physical impairments and no history of psychoactive or psychotropic substance use (screened by a previous anamnesis and a clinical examination). Moreover, participants were not included if they had less than 6 to 8 hours of sleep prior to the experiment and/or caffeine 48 hours prior to the experiment. All participants were made aware of the entire experimental protocol and signed a consent form before participating in this study. This study was approved by the Ethics Committee at Federal University of Rio de Janeiro.

### Stimuli

In order to minimize sensory interference, the experiment was performed in a sound and light-attenuated room. Participants were seated on a comfortable chair to minimize muscular artifacts, while EEG data was collected. During the visual task, lights were turned off and participants instructed to concentrate exclusively on the monitor screen. A 15” Samsung monitor was placed 50 cm in front of the participant. The visual stimulus was presented in the center of the screen by the ERP acquisition software with a visual angle of 1° x 1°, developed in DELPHI 5.0. To elicit the P300, all participants were presented with the same visual discrimination task, which employed the classical “oddball” paradigm [7]. In this paradigm, two stimuli are presented randomly, with one occurring infrequently. Participants were asked to discriminate targets (20%, infrequent) from non-targets or standard stimuli (80%, frequent). Target stimuli were defined as visual squares and non-targets as circles.

Participants were instructed to respond to target stimuli by pressing a button with their right index finger using a joystick (Quick Shot-Crystal CS4281, Quickshot®, USA). The RT needed to respond after each target stimulus was used as an index of motor performance. Participants’ reaction times were measured at each trial in milliseconds. Each participant received one block of 350 to 400 trials. In each block, there was a 95% chance of 1 to 4 non-target stimuli preceding a target stimulus and a 5% chance of 5 to 7 non-target stimuli preceding a target stimulus. Specifically, 100 target stimuli were presented in the block. The total number of stimuli presented (targets plus non-targets) varied between 350 and 400, and the ratio of target/non target stimuli was 1/4. Each stimulus appeared on the screen for 750 milliseconds with an inter-trial interval (onset to onset) of 1500 milliseconds.

### EEG data acquisition and processing

The International 10/20 system for electrodes was used with a 20-channel EEG system (Braintech-3000, EMSA-Medical Instruments, Brazil) [8]. The 20 electrodes were arranged in a nylon cap (ElectroCap Inc., Fairfax, VA, USA) yielding monopole derivations referred to linked earlobes. In addition, two 9 mm diameter electrodes were attached above and on the external corner of the right eye, in a bipolar electrode montage, for eye-movement (EOG) artifact monitoring. Impedance of EEG and EOG electrodes were kept under 5–10KΩ. The data acquired had total amplitude of less than 100 μV. The EEG signal was amplified with a gain of 22,000, analogically filtered between 0.01 Hz (high pass) and 100 Hz (low-pass), and sampled at 240 Hz. The software ERP Acquisition (Delphi 5.0), developed at the Brain Mapping and Sensorimotor Integration Laboratory, was employed to filter the raw data: notch (60 Hz), high-pass of 0.3 Hz and low-pass of 25 Hz.

To quantify reference-free data, both visual inspection and independent component analysis (ICA) were applied to remove possible sources of artifacts produced by the task. Data from individual electrodes exhibiting loss of contact with the scalp or high impedances (>10 kΩ), as well as data from single-trial epochs exhibiting excessive movement artifact (±100 μV) were discarded. Independent component analysis (ICA) was then applied to identify and remove any remaining artifacts after the initial visual inspection [9]. ICA is an information maximization algorithm that derives spatial filters by blind source separation of the EEG signals into temporally independent and spatially fixed components. Independent components resembling eye-blink or muscle artifacts were removed and the remaining components were then back-projected onto the scalp electrodes by multiplying the input data by the inverse matrix of the spatial filter coefficients derived from ICA using established procedures. The ICA-filtered data were then re-inspected for residual artifacts using the same rejection criteria described above [9].

### Statistical analysis

Participants were classified as “fast” or “slow” responders through PCA and DFA of ERP components and Reaction Time (RT) data. Principal components are the new variables generated through a special mathematical transformation performed on the original variables. This mathematical operation is available in several specialized statistical softwares. Each principal component is a linear combination of all original variables. For instance, a system with eight variables after transformation will have eight principal components. Each principal component will be written as a linear combination from eight original variables. In these combinations, each variable will have a different weight or importance. Within this context, PCA aimed at extracting from the ERP components, those components associated with greater variations in the sample.

In addition, DFA aimed at verifying the allocation of participants in groups related to the factors extracted by PCA. DFA is based on the variance maximization between groups in relation to the variance within the groups. The equation of the discriminant function is: Z = W_1_X_1_ + W_2_X_2_ + … + W_n_X_n,_ where Z = discriminant score, W_i_ = discriminant weight and X_i_ = independent variables. Student’s t-test was applied to statistically compare groups derived from PCA and confirmed by DFA .

## Results

We investigated early and late stages of information processing by assessing the latency, and amplitude of ERP components, including P200, N200, PMP and P300, directly comparing slow versus fast responding participants in the context of a visual oddball paradigm.

### Behavioral data

**Figure 1:**
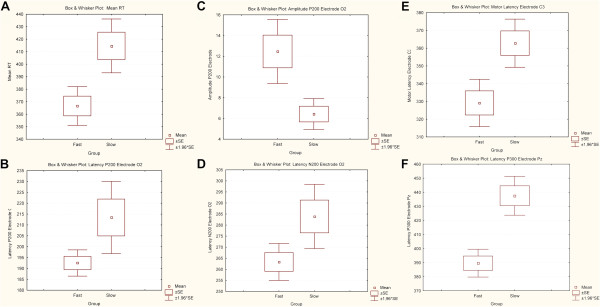
**Comparison among the amplitudes and latencies of P200 (electrode O2), and the latencies of N200 (electrode O2), PMP (electrode C3), P300 (electrode PZ) and the RT. A)** Comparison of mean RT between fast and slow groups. Significant difference, p = 0.001; **B)** Comparison of latencies of P200 waves of the electrode O2 between fast and slow groups. Significant difference, p = 0.015; **C)** Comparison of amplitudes of P200 waves of the electrode O2 between fast and slow groups. Significant difference, p = 0.008; **D)** Comparison of latencies of N200 waves of the electrode O2 between fast and slow groups. Significant difference, p = 0.018; **E)** Comparison of latencies of PMP waves of the electrode C3 between fast and slow groups. Significant difference, p = 0.003; **F)** Comparison of latencies of P300 waves of the electrode PZ between fast and slow groups. Significant difference, p = 0.001).

During the visual task, participants correctly responded to all presented target stimuli (100/100). The statistical analysis revealed that the RT of the fast group was significantly reduced when compared to the RT of the slow group (*p =* 0.001; mean fast = 366.583, SD fast = 27.301; mean slow = 414.625, SD slow = 31.075) (Figure 1A).

### ERP data

We assessed the early and late ERP components and the RT of the paradigm between slow and fast participants. We were specifically interested in comparing early and late ERP components representing early and late stages of information processing between slow and fast participants and, in determining the characteristics (latency, amplitude) and relationships of these components in the context of the paradigm.

**Figure 2:**
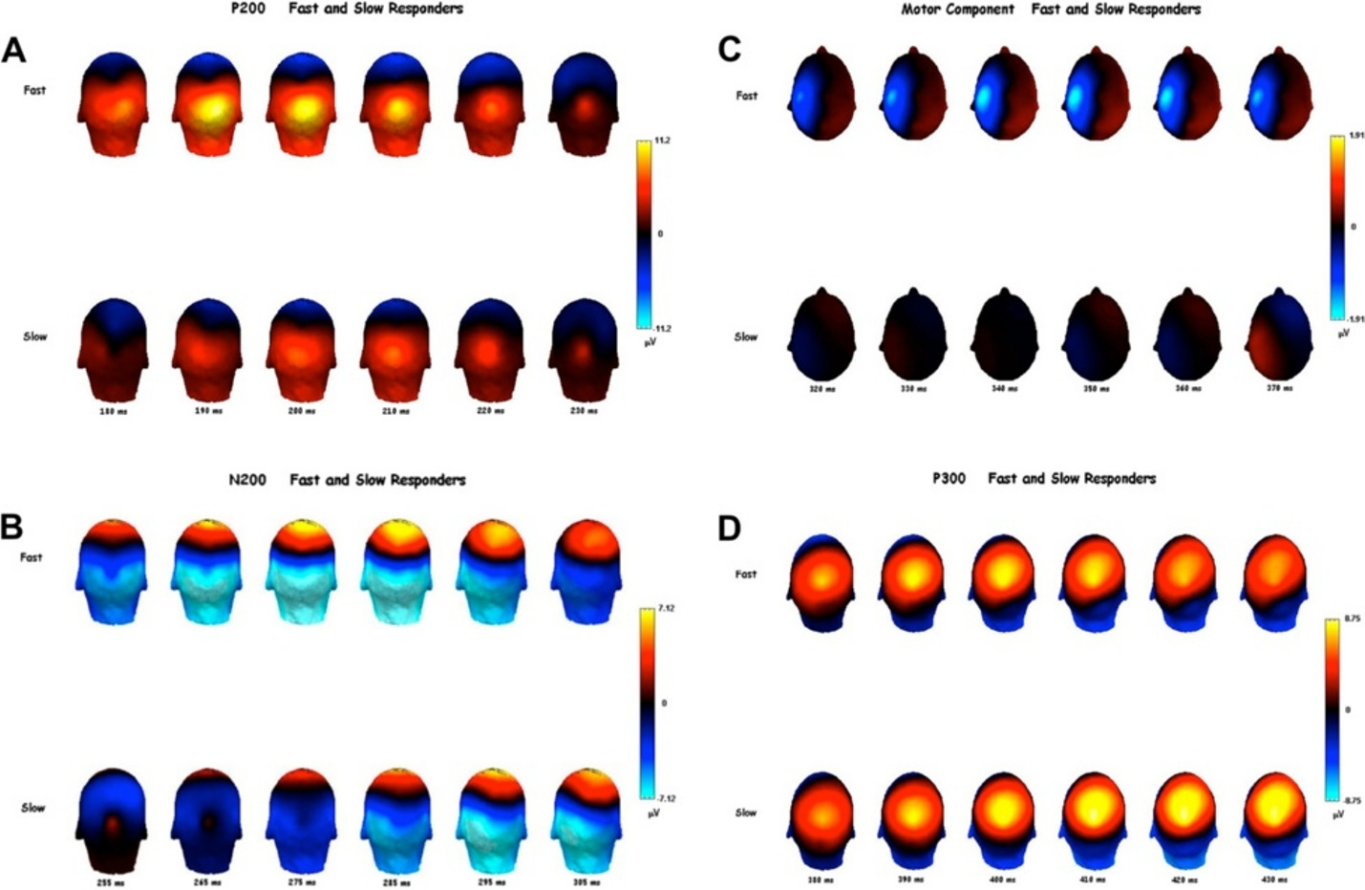
**Event-related potential plot for P200, N200, PMP and P300 waves. A)** Event-related potential plot for P200 wave between fast and slow groups; **B)** Event-related potential plot for N200 wave between fast and slow groups; **C)** Event-related potential plot for PMP wave between fast and slow groups; **D)** Event-related potential plot for P300 wave between fast and slow groups.

**Figure 3:**
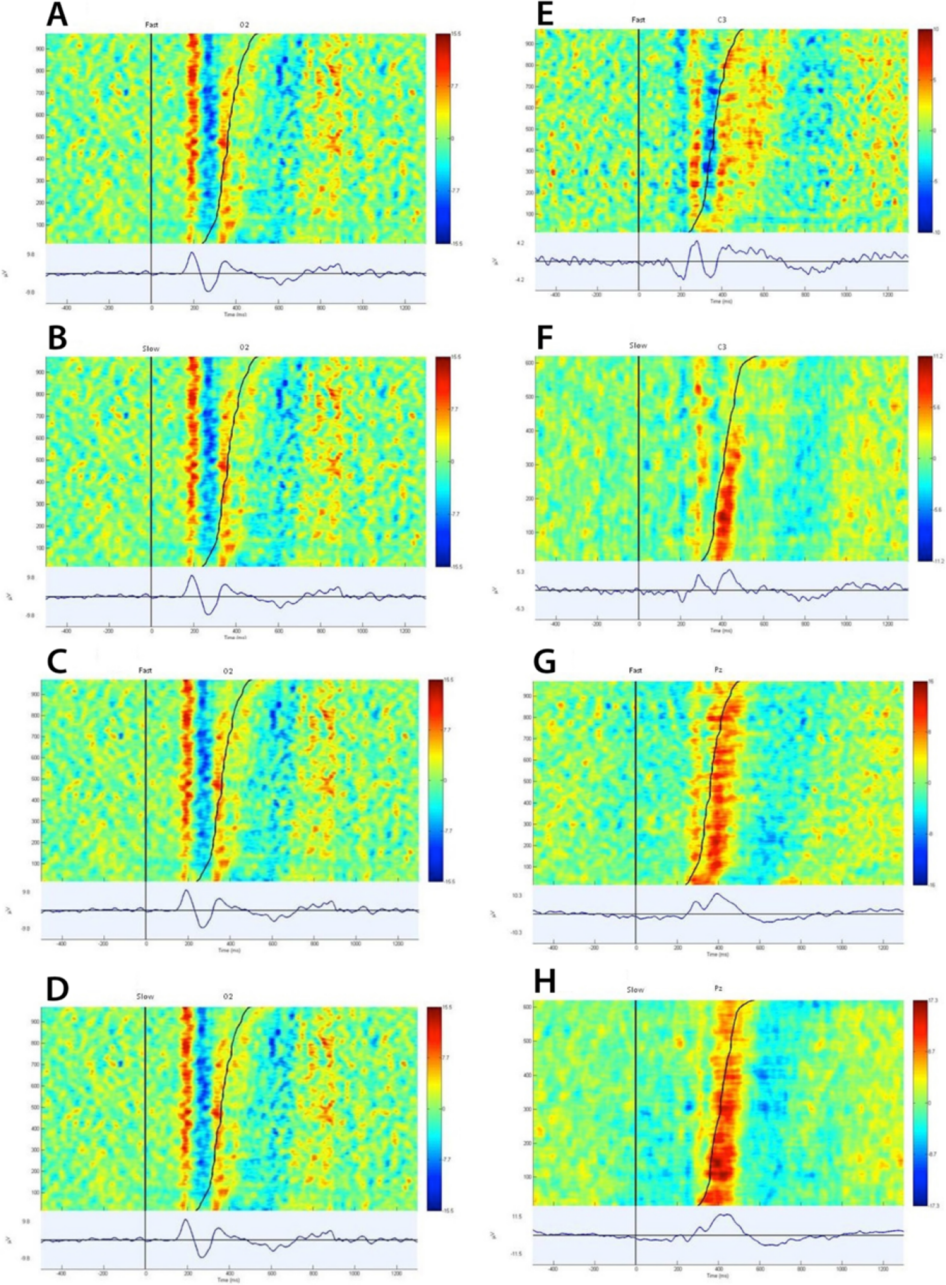
**Topographical distribution of event-related potential for P200, N200, PMP and P300 waves. (A) T**opographical distribution of event-related potential for P200 wave for fast group; **(B) T**opographical distribution of event-related potential for P200 wave for slow groups; **(C)** Topographical distribution of event-related potential for N200 wave for fast group; **(D)** Topographical distribution of event-related potential for N200 wave for slow group; **(E)** Topographical distribution of event-related potential for PMP wave for fast group; **(F)** Topographical distribution of event-related potential for PMP wave for slow group; **(G)** Topographical distribution of event-related potential for P300 wave fast group; **(H)** Topographical distribution of event-related potential for P300 wave slow group.

Statistical analysis revealed that the latency of the P200 component (observed in electrode O2) of the fast group was significantly reduced when compared to the slow group (*p =* 0.015; mean fast = 192.500, SD fast = 27.301; mean slow = 213.4375, SD slow = 31.075) (Figures 1B, 2A and 3A). In contrast, the amplitude of the P200 component (observed in electrode O2) of the fast group was significantly increased when compared to the slow group (*p =* 0.008; mean fast = 12.466, SD fast = 5.460; mean slow = 6.425, SD slow = 2.143) (Figure 1C).

Furthermore, for the fast group the latency of the N200 component (observed in electrode O2), the latency of the PMP component (observed in electrode C3) and the latency of the P300 component (observed in electrode Pz) were all significantly reduced when compared to the slow group (Figures 1D, 2B and 3B; 1E, 2C and 3C; and 1F, 2D and 3D, respectively; N200: *p =* 0.018; mean fast = 263.333, SD fast = 14.783; mean slow = 283.912, SD slow = 20.976; PMP: *p =* 0.003; mean fast = 329.166, SD fast = 23.508; mean slow = 362.812, SD slow = 19.568; P300: *p =* 0.001; mean fast = 389.583, SD fast = 17.478; mean slow = 437.500, SD slow = 19.955).

## Discussion

The statistical results demonstrate that the latencies of the P200 (O2 electrode), N200 (O2), PMP (C3) and P300 (Pz) components were significantly short in the group of fast responding participants; and that, at the same time, the amplitude of the P200 (O2) component was significantly higher in the same group of fast participants. These findings are discussed below, separately for each component, in more detail.

### Amplitude and latency of P200

The P200 component is a deflection with maximum positive amplitude between 150 and 250 ms [10]. It is associated with the allocation of attention [11] and with input secondary processing [12]. The higher the amplitude of a component, the higher the brain’s electrical activity required in the corresponding information processing stage. Bahramali and colleagues (1998) employed the auditive oddball paradigm to investigate the differences in ERP among participants with fast and slow RT. In contrast to our findings, their results revealed decreased P200 amplitude in the group of fast participants [13]. We propose that this divergence has occurred primarily because of two factors: first, due to the difference between both paradigms, auditory versus visual; and second, because of the criteria used to classify participants as slow or fast responders. While Bahramali and colleagues used only RT [13], we used the RT and the ERP components, specifically the P200, N200, P300 and the PPM. We suggest that the brain’s electrical activity required during input processing (high P200 Amplitudes) favors the subsequent components of the ERP as well as the RT, and thus contributes to the classification of the individual as fast.

On the other hand, and according with our findings, the results of Bahramali and colleagues also demonstrate a decrease in P200 latency in fast participants [13]. This allows us to assume that the input recording occurs earlier in fast participants, regardless of the model/stimuli (visual or auditive) or the criteria for classification of these individuals. In a previous study, Portella and colleagues observed a negative correlation trend (p = 0.22) (r = − 0.28) between the P200 amplitude and latency [14]. A larger sample would be more sensitive for the analysis of this correlation. However, we can infer that high amplitudes and latencies of the early P200 are able to interfere with the classification of individuals as fast or slow. In other words, when a higher brain electrical activity is required for the registration of visual inputs (P200 amplitude), probably related to the attention level, the second one will occur sooner during the recording period? (P200 latency).

### Latency of N200 (O2)

N200 has a maximum negative amplitude between 175 and 250 ms [10]. It is related to multiple neuronal processes associated with stimuli discrimination and classification [15–17]. Bahramali and colleagues also observed a short N200 latency [13], which allows us to conclude that the discrimination and classification of the inputs occur earlier in fast participants, regardless of the model/stimuli?? (visual or auditory). Based on the theory of vision pathways, in which the information occurs through parallel and serial pathways, and in which the end of one stage of information processing would be a prerequisite for the beginning of the next [18], it can be expected that in fast participants the N200 latency would be anticipated due to the precocity of P200 latency. In other words, the sooner the registration of visual input occurs (P200 latency), the earlier the discrimination and classification of this stimulus will take place (N200 latency). Such hypothesis is supported by the results of Portella and colleagues where a significantly positive correlation between the P200 and N200 latencies was verified [14].

### Latency of PMP (C3)

PMP has a negative amplitude that arises approximately 300 ms before the beginning of the movement with the peak around 100 ms or less [19]. It clearly occurs on the motor area contralateral to the movement [20]. It has been used to indicate the stage of ERP motor execution process [21, 22]. In the current study, we used a statistical analysis for ERP components as well as for RT in order to classify the participants as fast or slow. The results demonstrated early latencies of PMP in fast participants. The serial appearance of the processing stages of information ranging from the identification of the stimulus culminating in the motor execution or not [21], but is an important hypothesis which tries to elucidate such an outcome. The earlier study by Portella and colleagues reinforces this hypothesis [14]. This was supported by the positive correlation between the P200 latency (O2) and N200 latency (O2), PMP (C3), P300 (Pz) as well as the RT [14]. In other words, the sooner the visual information processing stages occur (P200 and N200), the earlier the motor execution processing (PMP) will take place. We therefore propose that the appearance of serial stages of processing of visual information, determine both the latency of the PPM as well as the classification of individuals as fast or slow responders.

### Latency of P300 (Pz)

P300 has a maximum positive amplitude between 250 and 600 ms [10]. It arises after the completion of a task related to stimuli differentiation, reflecting a reorganization of attention [23]. It is also related to the updating of working memory [11] and it is probably related to learning processes [24]. It has been suggested that this potential is elicited at the end of the cognitive processing in which the decision is made whether the stimulus is important or not [25]. Therefore, the P300 latency may reflect the time needed to interpret the stimulus as important [26].

In agreement with the findings of the current study, Bahramali and colleagues further verified a reduced P300 latency in fast participants in comparison with slow participants [13]. Thus, we can infer that regardless of the modality of inputs (visual or auditory) and by the “oddball” paradigm, participants present short P300 latency. Saito and colleagues hypothesized that P300 abnormalities may be caused by losses in the early stages of sensorial processing [27]. This seems consistent both with the theory of vision pathways [21] and with the result of the current study, in which the group of slow participants presented a delayed P200 and N200 latency. Thus, losses in the registration, discrimination and classification of sensorial inputs probably affect the reorganization of attention and working memory after the completion of a task related to stimuli differentiation.

### Reaction time (RT)

The motor reaction time employed in the current study comprises the interval of time between the onset of the target stimulus and pressing a button of a joystick. The results demonstrated that the group of fast participants presented a lower RT in relation to slow subjects. Jokeit & Makeig classified participants in two groups, namely fast and slow responders through a task related to an auditive “oddball” paradigm [28]. For the authors, the fast participants do not show a clear and conscious perception of the stimulus, while the slow participants inhibit their responses until the target stimulus is recognized and take a conscious decision to answer. Makeig and colleagues also classified participants in 2 groups, that is, fast and slow responders using the paradigm of visual “oddball” [29]. The authors suggest that the lower RT of fast participants can be triggered by the P3f component, which simultaneously appears in various regions of the brain so as to exert a pre-activation, thus favoring the RT in cases of decision to the motor action.

Several studies have reported higher ERP component latencies in Multiple Sclerosis (MS) patients [30, 31]. These results have been interpreted and supported by neuropsychological and neuroimaging evidences as an objective evidence of cognitive impairment, characterized by slow information processing as a result of impairments of the connections between cortical-subcortical and cortical-cortical structures [32–34]. This hypothesis could explain the greater latencies presented by the slow group, that is, even minimal impairments of these connections could affect information processing and hence, cognitive and motor processes.

Mei Li and colleagues studied sensorial and cognitive information processing in patients suffering from Parkinson’s disease through ERP and through the paradigms of visual oddball and S1 - S2 [35]. The results revealed a higher P200, N200, P300 latency and RT in the group of patients suffering from Parkinson’s disease in comparison to the control group. The authors suggest that the failure in inhibitory modulation in the early stages of sensorial information processing could explain the higher P200 and N200 latencies, and a deficit in the cognitive processing of such information could explain the higher P300 latency, and hence, the RT delay.

Hence, it can be concluded that i) impairments, even if minimal, in the early somatosensory information processing (P200 and N200) and the subsequent cognitive and motor stages (PMP, P300 and RT) can be detected by ERP, ii) the ERP research allows the evaluation of cognitive and sensorimotor dysfunctions in healthy old-aged participants and in neuropsychiatric patients (e.g., patients suffering from Parkinson’s disease and dementia), and iii) the various hypotheses which try to explain the smaller P200, N200, PMP and P300 latencies and the higher amplitude of P200 and the lower RT in the group of fast participants are not mutually exclusive, but rather complementary. Thus, the theory of vision pathways, especially its serial aspect; the learning ability and skill acquisition [36]; the P3f component, which simultaneously arises in several regions of the brain so as to exert a preparatory pre-activation [29]; losses in the connections between cortical-subcortical and cortical-cortical structures [30, 33, 34] and the failure in inhibitory modulation in the early stages of sensorial information processing and the deficit in cognitive processing of these information [36].
